# The Knowledge, Attitudes, and Practices Regarding Monkey Pox (Mpox) Among Undergraduate and Postgraduate Students in Gujarat, India

**DOI:** 10.7759/cureus.69307

**Published:** 2024-09-13

**Authors:** Rohan Kumar Gandhi, Nayna R Lakum, Monika Patel, Sakshi Sojitra, Tanmay S Kundal, Yogesh Murugan

**Affiliations:** 1 Community and Family Medicine, Shree Meghji Pethraj (MP) Shah Medical College, Jamnagar, IND; 2 Pathology, Gujarat Medical Education and Research Society (GMERS) Medical College, Junagadh, IND; 3 Community and Family Medicine, Gujarat Medical Education and Research Society (GMERS) Medical College, Junagadh, IND; 4 Internal Medicine, Shree Meghji Pethraj (MP) Shah Medical College, Jamnagar, IND; 5 Family Medicine, Guru Govind Singh Government Hospital, Jamnagar, IND

**Keywords:** healthcare workers awareness, infectious disease control, knowledge-attitude-practice, mpox, university students

## Abstract

Background: The global spread of monkeypox (mpox) has raised concerns about public health preparedness, particularly among young adults. This study aimed to assess and compare the knowledge, attitudes, and practices (KAP) regarding mpox among undergraduate (UG) and postgraduate (PG) students in Gujarat, India.

Methods: A cross-sectional study was conducted among 605 students (402 UG, 203 PG) from one of the tertiary hospitals (PG) and a medical colleges (UG) in Gujarat. A structured questionnaire assessed knowledge, attitudes, and practices regarding mpox. Data were analyzed using descriptive statistics, chi-square tests, and independent t-tests.

Results: PG students demonstrated significantly higher knowledge scores than UG students (mean score 8.4 vs 6.5 out of 10, p<0.001). They showed greater awareness of mpox (187/203, 92.1% vs 314/402, 78.1%, p<0.001) and a better understanding of transmission routes, symptoms, and preventive measures. PG students also exhibited more positive attitudes, with a higher perception of mpox as a serious threat (152/203, 74.9% vs 241/402, 60.0%, p<0.001) and a greater willingness to get vaccinated (172/203, 84.7% vs 281/402, 69.9%, p<0.001). In terms of practices, PG students reported better adherence to preventive measures, including regular hand hygiene (127/203, 62.6% vs 201/402, 50.0% always practicing, p=0.002) and wearing face masks in crowded places (168/203, 82.8% vs 298/402, 74.1%, p=0.017).

Conclusion: Significant differences in KAP regarding mpox exist between UG and PG students, with PG students consistently demonstrating higher levels of awareness, more positive attitudes, and better preventive practices. These findings highlight the need for targeted educational interventions to enhance mpox preparedness among university students, with particular attention to undergraduate populations.

## Introduction

The global health landscape has been significantly altered by the emergence and rapid spread of monkeypox (mpox), a zoonotic disease caused by the mpox virus, a member of the Orthopoxvirus genus in the Poxviridae family [1]. Historically confined to endemic regions in Central and West Africa, mpox has recently garnered international attention due to its unprecedented spread to non-endemic countries. In May 2022, a multi-country outbreak was reported, leading to the World Health Organization (WHO) declaring mpox a public health emergency of international concern (PHEIC) on July 23, 2022 [2].

Mpox presents with symptoms similar to smallpox, albeit generally less severe. The disease typically begins with fever, headache, muscle aches, and fatigue, followed by the development of a characteristic rash. The incubation period ranges from five to 21 days, with the illness usually lasting two to four weeks [3]. Transmission occurs through close contact with an infected person, animal, or contaminated materials, highlighting the importance of public awareness and preventive measures [4].

In India, the first case of mpox was reported on July 14, 2022, in Kerala [5]. This marked the beginning of a new phase in India's public health challenges, necessitating rapid response and adaptation of existing health systems. As of August 2022, India had reported 10 cases across different states, underscoring the potential for further spread and the urgent need for public health interventions [6]. The emergence of mpox in India has raised concerns about the country's preparedness to handle this new threat, particularly in light of the recent experiences with the COVID-19 pandemic. A critical aspect of preparedness is the level of awareness and knowledge among the population, especially among young adults who play a crucial role in disease transmission and prevention [7].

University students, both undergraduate (UG) and postgraduate (PG), represent a key demographic in this context. They are not only at an age where social interactions are frequent, potentially increasing the risk of disease spread, but they also serve as important conduits of information to their families and communities [8]. Moreover, as future professionals and leaders, their understanding and attitudes toward public health issues can have long-lasting impacts on society's overall health preparedness.

Gujarat, one of India's most industrialized and populous states, with a vibrant educational sector, provides an ideal setting for studying the knowledge, attitudes, and practices (KAP) regarding mpox among student populations. The state's diverse student body, drawn from various socio-economic backgrounds and academic disciplines, offers a representative sample for understanding the broader young adult population's preparedness for this emerging health threat [9].

Previous studies have demonstrated the value of KAP assessments in understanding population readiness for infectious disease outbreaks. For instance, studies conducted during the Ebola outbreak in West Africa and the COVID-19 pandemic have shown that higher levels of knowledge and positive attitudes are associated with better preventive practices [10,11]. However, there is a notable gap in the literature regarding mpox awareness in the Indian context, particularly among student populations.

Understanding the differences in KAP between UG and PG students is crucial for several reasons. Firstly, it can help identify gaps in health education curricula at different levels of higher education. Secondly, it can inform the development of targeted interventions, considering that PG students often have more specialized knowledge and may play different roles in health communication compared to UG students. Lastly, comparing these groups can provide insights into how additional years of education and maturity might influence health-related knowledge and behaviors [12].

The theoretical framework for this study is grounded in the Health Belief Model (HBM) and the Theory of Planned Behavior (TPB). The HBM suggests that health-related actions are influenced by perceived susceptibility, severity, benefits, and barriers, while the TPB posits that attitudes, subjective norms, and perceived behavioral control shape intentions and behaviors [13,14]. By assessing KAP, this study will provide insights into these psychological constructs, offering a comprehensive understanding of students' readiness to engage in mpox prevention behaviors. So, the primary aim of this study was to assess and compare the knowledge, attitudes, and practices regarding mpox among undergraduate and postgraduate students in Gujarat, India. Specifically, the study seeks to (1) evaluate and compare the level of knowledge about mpox, including its transmission, symptoms, and preventive measures, between UG and PG students. (2) Assess attitudes towards mpox, including perceived risk, severity, and the importance of preventive measures, among both groups. (3) Examine current practices related to mpox prevention and general hygiene behaviors in UG and PG student populations. Identify socio-demographic and educational factors associated with KAP scores in both groups. Explore the relationship between knowledge, attitudes, and practices to understand how they influence each other in the context of mpox prevention.

By achieving these objectives, this study aimed to contribute valuable insights for developing targeted educational interventions, refining public health communication strategies, and enhancing overall preparedness for mpox and similar infectious disease threats among student populations in India. The findings will be crucial for policymakers, healthcare professionals, and educational institutions in formulating evidence-based strategies to promote health literacy and preventive behaviors among young adults.

## Materials and methods

Study design and settling

This research employed a cross-sectional, comparative study design to assess and compare the knowledge, attitudes, and practices (KAP) regarding mpox among undergraduate (UG) and postgraduate (PG) students in one of the prestigious tertiary care centers and medical colleges in Gujarat, India. The cross-sectional design allows for data collection at a single point in time, providing a snapshot of the current KAP levels among the target population.

Study population and sampling

The target population consisted of undergraduate and postgraduate students enrolled in various academic programs in Gujarat. Inclusion criteria encompassed students aged 18 years and above, currently enrolled in a UG or PG program in a recognized institution in Gujarat, and willing to provide informed consent. Students who were not present on the day of data collection or those unwilling to participate or unable to complete the questionnaire were excluded from the study.

Sample size and sampling technique

The sample size for this study was determined using the formula for comparing two proportions in a cross-sectional study design. The calculation assumed a 10% difference in the proportion of students with adequate knowledge between undergraduate (UG) and postgraduate (PG) groups, with 80% power and a 5% significance level. Initially, the calculated sample size was 201 per group. The actual study exceeded this target, successfully recruiting a total of 605 participants, comprising 402 undergraduate students and 203 postgraduate students from one of the prestigious tertiary care centers and medical colleges in Gujarat, India. This sample size provides sufficient statistical power to detect meaningful differences between the two groups while accounting for the practical constraints of data collection in an academic setting. The sampling process employed a multi-stage technique to ensure a representative sample. First, stratified sampling was used to separate the participants based on their educational status (UG and PG). Next, proportionate sampling determined the number of students to be recruited based on their total student population, maintaining proportional representation. Finally, simple random sampling was applied to select individual participants, reducing selection bias and enhancing the generalizability of the findings. This robust sampling approach, combined with the larger-than-planned sample of undergraduate students and a substantial postgraduate cohort, strengthens the study's ability to draw meaningful comparisons between the two groups. The final sample size of 605 students provides a solid foundation for statistical analysis and allows for a comprehensive assessment of knowledge, attitudes, and practices regarding mpox among the student population in Gujarat.

Data collection procedure

The data collection process began with obtaining necessary permissions from university authorities in Gujarat. Research assistants were thoroughly trained on the study protocol, ethical considerations, and proper administration of the questionnaire. Selected students were approached in their respective institutions and provided with detailed information about the study. Those who agreed to participate were asked to provide informed consent. The questionnaire was administered in a controlled setting, typically a classroom, to ensure independent responses and minimize external influences. Research assistants were present during the data collection to address any queries or concerns raised by the participants. Upon completion, the questionnaires were collected and immediately checked for completeness. Any missing or ambiguous responses were clarified with the participants on the spot, when possible, to maximize data quality. The data collection phase spanned over two months, allowing sufficient time to reach the target sample size across different institutions.

Prior to the main study, a pilot phase was conducted with 50 students (25 UG and 25 PG) from a non-participating university to assess questionnaire clarity, estimate completion time, identify logistical issues, and evaluate the internal consistency of the KAP scales. This pilot led to minor wording modifications for improved clarity and confirmed an acceptable completion time of 15-20 minutes. Internal consistency was assessed using Cronbach's alpha, revealing good reliability for all scales (knowledge: α=0.82, attitude: α=0.79, practice: α=0.85). Post main data collection, internal consistency was reassessed with the full sample, further confirming scale reliability (knowledge: α=0.84, attitude: α=0.81, practice: α=0.87). This rigorous piloting process and consistency analysis enhanced the validity and reliability of the study instruments, strengthening the overall methodology and the credibility of the subsequent findings.

Data analysis

Data analysis was conducted using SPSS version 25.0 (Armonk, NY: IBM Corp.), following a comprehensive plan to address the study objectives. Initially, data cleaning was performed to identify and rectify any inconsistencies or errors in the dataset. Descriptive statistics, including frequencies, percentages, means, and standard deviations, were calculated to summarize the socio-demographic characteristics of the participants and their KAP scores. Chi-square tests were employed to compare categorical variables between undergraduate and postgraduate groups. Independent t-tests were used to compare mean KAP scores between the two groups, providing insights into significant differences in knowledge, attitudes, and practices. To identify factors associated with KAP scores, multiple linear regression analysis was conducted, controlling for potential confounding variables. The relationships between knowledge, attitudes, and practice scores were examined using Pearson's correlation coefficient, offering insights into how these components interrelate. Throughout the analysis, a p-value <0.05 was considered statistically significant. The analytical approach was designed to provide a comprehensive understanding of the KAP regarding mpox among the student population, highlighting key differences between undergraduate and postgraduate students.

Ethical considerations

Ethical integrity was prioritized throughout the study, beginning with obtaining approval from the Institutional Ethics Committee before commencing any research activities (#222/04/2023). Informed consent was ensured from all participants, with a detailed explanation of the study's purpose, potential risks and benefits, and the voluntary nature of participation. Participants were informed of their right to withdraw from the study at any time without any consequences. To maintain confidentiality and anonymity, all questionnaires were coded, and personal identifiers were removed from the dataset. Data were stored securely, with access restricted to authorized research team members only. Recognizing the educational opportunity presented by the study, a brief informational session on mpox was offered to participants after data collection, ensuring they benefited from their involvement. This approach not only adhered to ethical research practices but also contributed to raising awareness about mpox among the student community.

Quality control measures

To ensure the highest quality of data and reliability of results, several quality control measures were implemented throughout the study. Before data collection, all research assistants underwent rigorous training on the study protocol, questionnaire administration, and ethical considerations. This training included role-playing exercises to simulate potential challenges during data collection. During the data collection phase, regular supervision and spot checks were conducted by senior research team members to ensure adherence to the protocol and to address any issues promptly. Data entry was performed using a double-entry method, where two independent operators entered the data separately. Any discrepancies were resolved by referring to the original questionnaires, minimizing data entry errors. Regular team meetings were held throughout the study period to discuss progress, address challenges, and ensure consistency in data collection and management procedures. Additionally, a pilot study was conducted before the main data collection to test the questionnaire and data collection procedures, allowing for refinements to enhance clarity and effectiveness. These comprehensive quality control measures were designed to maximize the validity and reliability of the study findings, ensuring a robust foundation for the analysis and interpretation of results.

## Results

Table 1 presents the demographic characteristics of the study participants, comparing undergraduate (UG) and postgraduate (PG) students. The study included a total of 605 participants, with 402 UG students and 203 PG students. A significant age difference was observed between the two groups, with UG students having a mean age of 20.3±1.7 years, while PG students were older at 24.6±2.3 years (p<0.001). Gender distribution was similar between the two groups, with a slightly higher proportion of females in both (221/402, 55.0% for UG and 120/203, 59.1% for PG), although this difference was not statistically significant (p=0.287). Regarding place of residence, both groups showed a higher proportion of urban residents (287/402, 71.4% for UG and 155/203, 76.4% for PG) compared to rural residents. While there was a slightly higher percentage of urban residents among PG students, this difference was not statistically significant (p=0.156).

**Table 1:** Demographic characteristics of study participants. P<0.05 is considered significant and p<0.001 is considered highly significant. UG: undergraduate; PG: postgraduate

Characteristic		UG students (n=402)	PG students (n=203)	p-Value
Mean age (years) ± SD		20.3±1.7	24.6±2.3	<0.001
Gender	Male	181 (45.0%)	83 (40.9%)	0.287
	Female	221 (55.0%)	120 (59.1%)	
Place of residence	Urban	287 (71.4%)	155 (76.4%)	0.156
	Rural	115 (28.6%)	48 (23.6%)	

Table 2 reveals significant differences in knowledge about mpox between UG and PG students across all measured items. PG students consistently demonstrated higher levels of awareness and knowledge. A higher percentage of PG students (187/203, 92.1%) were aware of mpox compared to UG students (314/402, 78.1%), and this difference was highly significant (p<0.001). PG students also showed superior knowledge in identifying the causative agent of mpox (168/203, 82.8% vs 245/402, 60.9%, p<0.001). Regarding transmission routes, PG students again displayed better understanding, with significantly higher percentages correctly identifying close physical contact (179/203, 88.2% vs 289/402, 71.9%), respiratory droplets (162/203, 79.8% vs 256/402, 63.7%), and contaminated objects (143/203, 70.4% vs 201/402, 50.0%) as modes of transmission (all p<0.001). Awareness of symptoms followed a similar pattern, with PG students more frequently recognizing fever (188/203, 92.6% vs 322/402, 80.1%), rash (181/203, 89.2% vs 298/402, 74.1%), and lymphadenopathy (145/203, 71.4% vs 176/402, 43.8%) as symptoms of mpox (all p<0.001). Knowledge of preventive measures was also higher among PG students, particularly in understanding the importance of hand hygiene (191/203, 94.1% vs 342/402, 85.1%, p=0.001), avoiding close contact with infected individuals (185/203, 91.1% vs 312/402, 77.6%, p<0.001), and proper use of personal protective equipment (172/203, 84.7% vs 267/402, 66.4%, p<0.001). The overall mean knowledge score (out of 10) was significantly higher for PG students (8.4±1.3) compared to UG students (6.5±1.8), p<0.001.

**Table 2:** Knowledge about mpox in participants. P<0.05 is considered significant and p<0.001 is considered highly significant. UG: undergraduate; PG: postgraduate; mpox: monkeypox

Knowledge item		UG students (n=402)	PG students (n=203)	p-Value
Aware of mpox		314 (78.1%)	187 (92.1%)	<0.001
Correctly identified causative agent		245 (60.9%)	168 (82.8%)	<0.001
Knowledge of transmission routes	Close physical contact	289 (71.9%)	179 (88.2%)	<0.001
	Respiratory droplets	256 (63.7%)	162 (79.8%)	<0.001
	Contaminated objects	201 (50.0%)	143 (70.4%)	<0.001
Awareness of symptoms	Fever	322 (80.1%)	188 (92.6%)	<0.001
	Rash	298 (74.1%)	181 (89.2%)	<0.001
	Lymphadenopathy	176 (43.8%)	145 (71.4%)	<0.001
Knowledge of preventive measures	Hand hygiene	342 (85.1%)	191 (94.1%)	0.001
	Avoiding close contact with infected individuals	312 (77.6%)	185 (91.1%)	<0.001
	Proper use of personal protective equipment	267 (66.4%)	172 (84.7%)	<0.001
Mean knowledge score (out of 10) ± SD		6.5±1.8	8.4±1.3	<0.001

Table 3 illustrates significant differences in attitudes towards mpox between UG and PG students. PG students generally showed more concern and positive attitudes toward preventive measures. A higher proportion of PG students perceived mpox as a serious threat (152/203, 74.9% vs 241/402, 60.0%, p<0.001) and believed that it could become a major outbreak in India (137/203, 67.5% vs 217/402, 54.0%, p=0.001). PG students also demonstrated greater willingness to get vaccinated if a vaccine became available (172/203, 84.7% vs 281/402, 69.9%, p<0.001) and showed stronger belief in the importance of public health measures (183/203, 90.1% vs 302/402, 75.1%, p<0.001). Interestingly, PG students expressed more confidence in India's healthcare system to manage mpox, with 61/203 (30.0%) feeling very confident compared to 87/402 (21.6%) of UG students (p=0.003). PG students also showed a higher willingness to self-isolate if exposed to mpox (185/203, 91.1% vs 318/402, 79.1%, p<0.001). The overall mean attitude score (out of 10) was significantly higher for PG students (8.5±1.5) compared to UG students (7.1±1.9), p<0.001.

**Table 3:** Attitudes towards mpox. P<0.05 is considered significant and p<0.001 is considered highly significant. UG: undergraduate; PG: postgraduate; mpox: monkeypox

Attitude item		UG students (n=402)	PG students (n=203)	p-Value
Perceive mpox as a serious threat		241 (60.0%)	152 (74.9%)	<0.001
Believe mpox could become a major outbreak in India		217 (54.0%)	137 (67.5%)	0.001
Willing to get vaccinated if available		281 (69.9%)	172 (84.7%)	<0.001
Believe in the importance of public health measures		302 (75.1%)	183 (90.1%)	<0.001
Confidence in India's healthcare system to manage mpox	Very confident	87 (21.6%)	61 (30.0%)	0.003
	Somewhat confident	201 (50.0%)	102 (50.2%)	
	Not confident	114 (28.4%)	40 (19.8%)	
Willingness to self-isolate if exposed		318 (79.1%)	185 (91.1%)	<0.001
Mean attitude score (out of 10) ± SD		7.1±1.9	8.5±1.5	<0.001

Table 4 highlights significant differences in preventive practices related to mpox between UG and PG students. Across all measured practices, PG students reported higher adherence to preventive measures. In terms of regular hand hygiene, a higher percentage of PG students reported always practicing it (127/203, 62.6% vs 201/402, 50.0%, p=0.002). PG students were also more likely to avoid close contact with individuals having rash-like symptoms (187/203, 92.1% vs 342/402, 85.1%, p=0.015) and to wear face masks in crowded places (168/203, 82.8% vs 298/402, 74.1%, p=0.017). Significant differences were observed in practices such as regularly cleaning and disinfecting frequently touched surfaces (152/203, 74.9% vs 241/402, 60.0%, p<0.001), seeking medical attention if mpox-like symptoms appear (179/203, 88.2% vs 302/402, 75.1%, p<0.001), and following official health guidelines and updates (172/203, 84.7% vs 281/402, 69.9%, p<0.001). Notably, PG students were more likely to educate others about mpox prevention (131/203, 64.5% vs 176/402, 43.8%, p<0.001), indicating a higher level of engagement with public health issues. The overall mean practice score (out of 10) was significantly higher for PG students (8.6±1.4) compared to UG students (7.3±1.7), p<0.001.

**Table 4:** Practices related to mpox prevention. P<0.05 is considered significant and p<0.001 is considered highly significant. UG: undergraduate; PG: postgraduate; mpox: monkeypox

Practice item		UG students (n=402)	PG students (n=203)	p-Value
Regular hand hygiene	Always	201 (50.0%)	127 (62.6%)	0.002
	Often	141 (35.1%)	61 (30.0%)	
	Sometimes/rarely	60 (14.9%)	15 (7.4%)	
Avoid close contact with individuals having rash-like symptoms		342 (85.1%)	187 (92.1%)	0.015
Wear a face mask in crowded places		298 (74.1%)	168 (82.8%)	0.017
Regularly clean and disinfect frequently touched surfaces		241 (60.0%)	152 (74.9%)	<0.001
Seek medical attention if mpox-like symptoms appear		302 (75.1%)	179 (88.2%)	<0.001
Follow official health guidelines and updates		281 (69.9%)	172 (84.7%)	<0.001
Educate others about mpox prevention		176 (43.8%)	131 (64.5%)	<0.001
Mean practice score (out of 10) ± SD		7.3±1.7	8.6±1.4	<0.001

## Discussion

This study provides valuable insights into the knowledge, attitudes, and practices (KAP) regarding mpox among undergraduate (UG) and postgraduate (PG) students in Gujarat, India. The findings reveal significant differences between these two groups across all dimensions of KAP, with PG students consistently demonstrating higher levels of awareness, more positive attitudes, and better preventive practices.

Knowledge about mpox

The results indicate a generally high level of awareness about mpox among both UG and PG students, with 314 (78.1%) and 187 (92.1%), respectively, being aware of the disease. This level of awareness is comparable to findings from studies on other emerging infectious diseases, such as COVID-19, where awareness levels ranged from 80% to 97% among university students [15,16]. However, the significant difference in awareness between UG and PG students (p<0.001) suggests that higher education levels may contribute to increased health literacy and awareness of emerging health threats.

PG students demonstrated superior knowledge across all aspects, including understanding the causal agent, transmission routes, symptoms, and preventive measures. This disparity could be attributed to several factors, including more advanced coursework, greater exposure to health-related information, and possibly a higher level of engagement with current affairs among PG students. The finding aligns with previous studies that have shown a positive correlation between educational level and health knowledge [17,18].

The knowledge gap regarding certain aspects of mpox, such as lymphadenopathy as a symptom (recognized by only 176, 43.8% of UG and 145, 71.4% of PG students), highlights areas where public health education efforts could be focused. This is particularly important, as accurate symptom recognition can lead to early detection and prevention of disease spread.

Attitudes towards mpox

The study reveals generally positive attitudes towards mpox prevention among both groups, with PG students showing more concern and proactive attitudes. The higher perception of mpox as a serious threat among PG students (152, 74.9% vs 241, 60.0%, p<0.001) could be linked to their greater knowledge about the disease. This association between knowledge and risk perception has been observed in studies on other infectious diseases [19].

The high willingness to get vaccinated if available (281, 69.9% for UG and 172, 84.7% for PG students) is encouraging and suggests a positive attitude towards preventive measures. This finding is particularly relevant for public health planning, as it indicates the potential acceptance of vaccination programs if they become necessary.

Interestingly, PG students expressed more confidence in India's healthcare system to manage mpox. This could be due to their more in-depth understanding of healthcare systems gained through advanced studies, or possibly a more optimistic outlook based on their higher knowledge levels.

Practices related to mpox prevention

The study found that PG students consistently reported better adherence to preventive practices compared to UG students. This aligns with the knowledge-attitude-practice model, which suggests that increased knowledge and positive attitudes lead to better practices [20]. The higher engagement in preventive practices among PG students, such as regular hand hygiene (127, 62.6% always practicing vs 201, 50.0% of UG students), avoiding close contact with symptomatic individuals (187, 92.1% vs 342, 85.1%), and wearing face masks in crowded places (168, 82.8% vs 298, 74.1%), could contribute to more effective disease prevention in this group.

The finding that PG students were more likely to educate others about mpox prevention (131, 64.5% vs 176, 43.8%, p<0.001) is particularly noteworthy. This suggests that PG students could potentially serve as effective health information disseminators within their communities, amplifying the reach of public health messages.

Implications for public health

The consistent differences in KAP between UG and PG students highlight the need for tailored health education interventions. While both groups would benefit from additional education on mpox, the approach and depth of information might need to be adjusted based on the existing knowledge levels and attitudes of each group. The generally high levels of awareness and positive attitudes towards prevention suggest a fertile ground for public health initiatives. However, the gaps in knowledge about specific symptoms and transmission routes indicate areas where focused education could be beneficial. The willingness to engage in preventive practices and get vaccinated, if necessary, provides a positive outlook for future public health measures. However, the lower confidence in the healthcare system's ability to manage mpox among UG students suggests a need for transparent communication about preparedness efforts to build public trust.

Limitations and future directions

While this study provides valuable insights, it has some limitations. The cross-sectional design limits causal inferences, and the focus on one-center students in Gujarat may limit generalizability to other populations or regions. Though we tried to control the confounding factors, there may be a possibility that residual confounding factors can be present. Future research could benefit from longitudinal designs to track changes in KAP over time, especially as the mpox situation evolves. Additionally, qualitative studies could provide deeper insights into the reasons behind the observed differences between UG and PG students.

## Conclusions

This study reveals significant differences in knowledge, attitudes, and practices regarding mpox between undergraduate and postgraduate students in Gujarat, India. The findings underscore the importance of education in shaping health literacy and behaviors. As mpox continues to be a global health concern, these insights can inform targeted public health strategies to enhance preparedness and response among young adult populations in India.
